# Diagnostic Approach to Elevated Liver Function Tests during Pregnancy: A Pragmatic Narrative Review

**DOI:** 10.3390/jpm13091388

**Published:** 2023-09-16

**Authors:** Elton Dajti, Angelo Bruni, Giovanni Barbara, Francesco Azzaroli

**Affiliations:** 1 IRCCS Azienda Ospedaliero-Universitaria di Bologna, European Reference Network on Hepatological Diseases (ERN RARE-LIVER), 40138 Bologna, Italy; angelo.bruni3@studio.unibo.it (A.B.); giovanni.barbara@unibo.it (G.B.); francesco.azzaroli@unibo.it (F.A.); 2 Department of Medical and Surgical Sciences (DIMEC), University of Bologna, 40138 Bologna, Italy

**Keywords:** pregnancy, liver disease, liver enzymes, intrahepatic cholestasis of pregnancy, pre-eclampsia, elastography

## Abstract

Liver disease is not uncommon during pregnancy and is associated with increased maternal and fetal/neonatal morbidity and mortality. Physiological changes during pregnancy, including a hyperestrogenic state, increase in circulating plasma volume and/or reduction in splanchnic vascular resistance, and hemostatic imbalance, may mimic or worsen liver disease. For the clinician, it is important to distinguish among the first presentation or exacerbation of chronic liver disease, acute liver disease non-specific to pregnancy, and pregnancy-specific liver disease. This last group classically includes conditions such as hyperemesis gravidarum, intrahepatic cholestasis of pregnancy, liver disorders associated with the pre-eclampsia spectrum, and an acute fatty liver of pregnancy. All of these disorders often share pathophysiological mechanisms, symptoms, and laboratory findings (such as elevated liver enzymes), but a prompt and correct diagnosis is fundamental to guide obstetric conduct, reduce morbidity and mortality, and inform upon the risk of recurrence or development of other chronic diseases later on in life. Finally, the cause of elevated liver enzymes during pregnancy is unclear in up to 30–40% of the cases, and yet, little is known on the causes and mechanisms underlying these alterations, or whether these findings are associated with worse maternal/fetal outcomes. In this narrative review, we aimed to summarize pragmatically the diagnostic work-up and the management of subjects with elevated liver enzymes during pregnancy.

## 1. Introduction

Liver disease is not uncommon during pregnancy, and it is associated with increased maternal and fetal/neonatal morbidity and mortality [1]. A prompt and correct diagnosis of liver disease is pivotal for the clinician in order to timely initiate treatment, guide obstetric conduct, and improve maternal and fetal outcomes. The most common causes of liver involvement are disorders unique to pregnancy (estimated prevalence of 3%) [2], and encompass hyperemesis gravidarum (HG), intrahepatic cholestasis of pregnancy (ICP), conditions associated with gestational hypertensive disorders, such as pre-eclampsia (PE), HELLP (hemolysis, elevated liver enzymes, and low platelet count) syndrome, and an acute fatty liver of pregnancy (AFLP). Other causes of liver dysfunction are represented by new onset/first manifestation of a liver or biliary disease not specific to pregnancy (i.e., viral, autoimmune, and cholelithiasis) or exacerbation of the pre-existing chronic liver disease.

Abnormal liver function tests (LFTs) are a hallmark of the diagnosis of liver disease, yet their interpretation during pregnancy is not simple. Due to physiological changes and hemodilution, transaminases (especially alanine aminotransferase, ALT) [3] and gamma-glutamyl transpeptidase (GGT) are about 20% lower in pregnant women when compared with laboratory reference ranges [4] while alkaline phosphatase (ALP) increases due to placental contribution. Moreover, LFTs are not routinely recommended during pregnancy or prenatal testing, so the true prevalence of elevated LFTs or whether the pregnancy-specific upper limit of normal (ULN) values should be used is currently unknown. Indeed, a recent study found that up to 12% of pregnant women without known liver disease had ALT ≥ 25 IU/L at labor, and almost half of the patients with elevated LFTs did not have a clinical diagnosis of liver disease or have liver tests ever checked as part of routine pregnancy care [5]. So, the true burden of liver dysfunction and its prognostic impact on pregnancy outcomes remain largely unknown.

**Figure 1:**
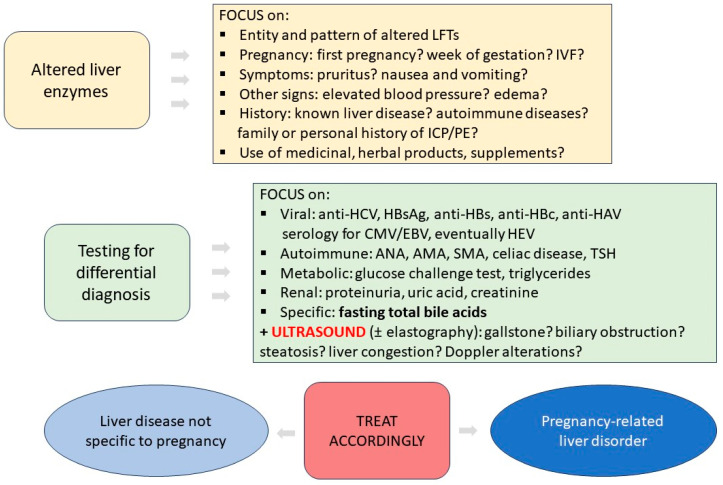
Diagnostic work-up of altered liver function tests during pregnancy.

In this narrative review, we aimed to summarize pragmatically the diagnostic work-up (Figure 1) and the management of subjects with elevated LFTs during pregnancy.

## 2. Acute and Chronic Liver Diseases Not Specific to Pregnancy

### 2.1. Viral Liver Disease

An estimated 4.5 million women with chronic HBV infection give birth annually, but the prevalence is highest in Africa and western Pacific regions [6]. It is recommended that HBsAg+ pregnant women undergo repeated testing (including LFTs, HBeAg, HBeAb, and HBV DNA levels) with a dual aim [7,8]: (i) reduce mother-to-child transmission (MTCT) of HBV, which depends on HBV-DNA levels and HBeAg status; (ii) monitor for any exacerbation of the maternal disease, as flares with raised transaminase levels can occur during or especially after pregnancy (5–25% of the cases) [9,10]. Current guidelines recommend initiation of prophylaxis antiviral treatment (preferred tenofovir) around the 24th–28th week of pregnancy in patients with HBV-DNA levels > 200,000 IU/mL of HBsAg levels > 4log10 IU/m [7,11]. If the patient was already on antiviral treatment before pregnancy, this should be continued, and switching from entecavir to tenofovir is recommended [7]. On the other hand, acute HBV infection is rare, presents a similar clinical course to that of non-pregnant females, and is associated with a lower rate of seroconversion [12,13].

Prevalence of HCV infection among pregnant women is presumed to be 1–3% [14,15], but global estimates are lacking [6]. Differently from HBV, pregnancy does not affect the course of HCV infection, so no specific monitoring is needed [8]. Pregnant women with HCV should be informed about an increased risk of pre-term birth [16] and ICP [17], and offered treatment with the new direct-acting antivirals after delivery, as little data are available on their safety during pregnancy. The risk of MCTC is around 3–5% [18], of which 40% is probably transmitted in utero [19], and infants should be tested for anti-HCV after 18 months of age, as passive transfer of antibodies usually occurs [20]. Acute and fulminant HCV infection during pregnancy is anectodical.

The HEV seroprevalence among pregnant women is estimated at 4–16% [21,22] (up to 30–50% in endemic areas such as Africa [23]). Unfortunately, HEV can be particularly virulent and severe in pregnancy, conveying a substantially higher rate of maternal and fetal morbidity and mortality than in non-pregnant women [22,24,25]; the risk of acute infection is also higher than with other hepatitis viruses [6,26]. The clinical manifestations range from mild sub-clinical disease and self-limiting acute infection to fulminant hepatic failure (in up to 60% of the cases) [6,8,26]. Management is mainly supportive, as ribavirin use is precluded because of the teratogenic effects [6,8].

Regarding minor hepatotropic viruses, HSV hepatitis is a very rare but life-threatening condition, leading to transplantation or death in 75% of the cases [27]. Pregnancy is a risk factor for HSV hepatitis and accounts for up to one-fourth of the cases [27]. Median AST or ALT values are 5000 IU/L, and coagulopathy and hepatic encephalopathy develop in up to 80% of the cases [27]. Empirical treatment with intravenous acyclovir should be started as soon as HSV hepatitis is suspected [8].

### 2.2. Autoimmune Liver Disease

Autoimmune hepatitis (AIH) is a chronic inflammatory autoimmune disease that primarily affects women with a bimodal age pattern at presentation [28]. Pregnancy in the context of AIH is a critical issue, as it can lead to obstetric complications such as a pre-term birth, a miscarriage, and perinatal morbidity, especially if cirrhosis is present [29,30,31,32]. As for maternal outcomes, AIH activity usually decreases during pregnancy [32,33,34], so the immunosuppressive therapy should be titrated to the lowest effective dose, but flares can occur in 30% (13–55%) of the cases, especially in the postpartum period [31,32,35,36]. Prednisone and azathioprine are considered safe in pregnancy and represent the mainstay of AIH treatment in pregnant women; on the other hand, treatments such as mycophenolate mofetil and tacrolimus are contraindicated due to harmful effects on the fetus [8,28]. Finally, AIH is very rarely diagnosed during pregnancy, but, like any other autoimmune disease, it may notably manifest in the postpartum period [28,37].

As for AIH, women with primary biliary cholangitis (PBC) have stable or reduced activity during pregnancy in 70% of the cases, but disease activation at postpartum develops in up to 60–70% of the cases [38,39,40]. However, pruritus may develop or worsen in a considerable rate of cases [38,40], and some patients may have a superimposed ICP. Ursodeoxycholic acid is safe during pregnancy and lactation, while data on fibrates and obeticholic acid are limited [8]. Notably, a minority of new PBC diagnoses are made during pregnancy [38], so this condition should always be considered when investigating altered LFTs in pregnant women.

The clinical picture for primary sclerosing cholangitis (PSC) is similar to PBC, if not more complicated, due to the paucity of data available in published literature, difficult interpretation of the data because of the concomitant inflammatory bowel disease in many patients, and lack of approved treatments for PSC [8,41,42,43].

## 3. Pregnancy-Related Liver Disorders

The main findings on liver function tests during pregnancy-related liver diseases are summarized in Table 1.

### 3.1. Hyperemesis Gravidarum

Hyperemesis gravidarum typically occurs during the first trimester of pregnancy and is defined as persistent, excessive, and intractable vomiting, resulting in dehydration, electrolytic imbalance/ketosis, and a weight loss of greater than 5% of the pre-pregnancy weight [44]. Liver enzymes (AST and ALT) are elevated in about 8–15% of the overall cases [44,45], and in up to 40–50% of the hospitalized patients with HG [46,47]; but the rise is usually mild (median values of 50 IU/L [46], usually a 2–5-fold × ULN), while jaundice is rare. These findings are likely related to starvation, correlate with disease severity [45,47], and usually return to normal values after successful management of HG with intravenous rehydration, vitamin supplementation, antiemetics, and gradual reintroduction of oral intake [1]. Abnormal LFTs that are much more pronounced than the above-mentioned values or that do not improve on the resolution of vomiting should prompt further diagnostic investigation.

### 3.2. Intrahepatic Cholestasis of Pregnancy

Intrahepatic cholestasis of pregnancy is the most common pregnancy-related liver disease (overall prevalence of 0.1–1.5%) [2], with a complex pathophysiology that includes multiple genetic, hormonal, and environmental components [48,49,50]. It usually occurs in the second or third trimester (80% after the 30th week) [8], and the diagnosis is established with the presence of pruritus and elevated serum bile acids; the most commonly used threshold for this purpose is 10 µmol/L (or 19 µmol/L for nonfasting measurements) [1,8,51,52]. Maternal complications of ICP are minimal, even though severe symptomatic pruritus may reduce the quality of life [51]. The main risks of ICP are related to the fetus, with an increased risk of a pre-term birth, meconium-stained amniotic fluid, neonatal respiratory depression, and intrauterine fetal demise [51,53,54,55].

Elevated levels of LFTs, namely transaminases, are a very common (17–80%) [1,44,56] finding in patients with ICP, with mean values of 148 IU/L for ALT and 105 IU/L for AST in >2000 patients with ICP [53] (range of 1.5–8-fold increase × ULN) [44]. However, elevated LFTs are not considered a diagnostic criterion anymore [51], especially because they do not correlate with ICP severity and bear limited prognostic significance, if any at all. In fact, in an individual patient data meta-analysis of 5269 ICP cases [53], only total serum bile acid levels were associated with stillbirths, while the AUROCs for the prediction of negative neonatal/fetal outcomes were all <0.6 for ALT and AST levels.

The first line of treatment for ICP is ursodeoxycholic acid (UDCA), which results in a biochemical response and improvement of maternal symptoms in most (75%) of the cases [44,57,58]. However, no data are available to assess whether the rate of improvement in AST/ALT levels correlates with clinical outcomes, or whether a treat-to-target strategy is to be pursued and used to titrate the UDCA dose.

### 3.3. Hypertension-Related Liver Disease during Pregnancy

Pre-eclampsia (PE) is a complex multisystem disease, diagnosed with a sudden onset of hypertension (at >20 weeks of gestation) and at least one other associated complication, either maternal (proteinuria, acute kidney injury, AST or ALT > 40 IU/L, neurological symptoms, hematological abnormalities, coagulation imbalance, and cardiopulmonary) or related to uteroplacental dysfunction (i.e., fetal growth restriction, placental abruption, and angiogenic imbalance) [59]. This condition is one of the most severe complications of pregnancy and a leading cause of maternal and perinatal morbidity and mortality [60]. The risk of complications becomes even higher when seizures develop (eclampsia), or when the triad of hemolysis (±LDH ≥ 600 IU/L), elevated liver enzymes (AST > 70 IU/L, and a low platelet count (<150,000/mm^3^) (HELLP syndrome) is present.

From a clinical perspective, all patients with PE are at risk of rapid progression and severe disease regardless of the timing of onset [59,61]. Yet, from a mechanistic and pathophysiological point of view, PE can be classified based on the gestational age at the time of delivery (pre-term, term, or postpartum, or early vs. late-late onset), reflecting a possible underlying difference in etiology [62]. In pre-term PE, abnormal placentation (inadequate trophoblast invasion and spiral artery remodeling) seems to play a central role in the development of syncytiotrophoblast stress and placental hypoperfusion [63,64]. This could explain why the accuracy of early pregnancy predictive models, which are based on biomarkers of abnormal placentation, is high for pre-term PE (sensitivity of 75–90%), but far from optimal for term PE (<50%) [63,64]. In term PE, syncytiotrophoblast stress seems to develop later in gestation, possibly related to compression of the chronic villus when there is insufficient space for the larger placenta in late pregnancy and syncytiotrophoblast senescence associated with premature placental aging; maternal metabolic and cardiovascular function (insulin resistance, metabolic syndrome, and mitochondrial and lipid dysfunction) seems to contribute to this altered placental metabolic dysfunction that can escalate to late-onset PE [65,66].

In patients with PE, altered LFTs usually reflect vasoconstriction of the hepatic vascular bed and hepatic injury due to the diffuse microangiopathy (ischemia and oxidative stress in hepatic sinusoids and hemolysis) [2,62], and bear both diagnostic and prognostic importance.

From a diagnostic point of view, elevated AST/ALT levels are found in 20–50% of the patients [2,44], with mean values of 40–100 IU/L [67,68] (usually 2–5-fold increase × ULN), and as stated above, contribute to the establishment of a PE diagnosis in the presence of hypertension. Patients with HELLP usually present mean AST/ALT values of 300–500 IU/L [69,70] that can go up to 30 × ULN, and not infrequently (15%), jaundice [71].

From a prognostic point of view, elevated liver enzymes are an important and independent risk factor for the development of unfavorable maternal outcomes [62,72,73]. Therefore, transaminases are included in most of the scores used in clinical practice to stratify for the risk of maternal complications in patients with PE, such as PREP-S [74] and fullPIERS [75]. However, LFTs are a suboptimal predictor of fetal/neonatal outcomes, especially after adjustment for gestational age [76].

Finally, some studies have suggested that elevated LFTs in early pregnancy can predict the future development of PE and HELLP [77,78,79]; still, most of these studies do not distinguish between pre-term/early PE and term/late PE. It seems that this association is independent of other confounders such as age, body mass index, or the presence of diabetes. However, whether this hypertransaminasemia reflects a dysmetabolic profile of the mother, a known risk factor for PE, especially for term PE, or is an early marker of liver and systemic involvement in patients with PE is still to be established.

### 3.4. Acute Fatty Liver of Pregnancy

An acute fatty liver of pregnancy (AFLP) is a rare condition that occurs in the third trimester or at postpartum in 0.005–0.01% of pregnancies [80], and it represents an obstetric emergency. Its pathogenesis is not well understood, but disorders of fatty acid oxidation have been shown to contribute in some patients [81]. The clinical picture often overlaps with severe forms of PE and HELLP syndrome,1 but then complications typical of acute liver failure of any etiology develop [82]. The Swansea criteria are the most commonly used diagnostic criteria, but they are not without limitations [82]. Levels of transaminases and/or bilirubin are included among these criteria and are almost always above the normal range, with AST/ALT levels of 300–1000 IU/L (median of 300–310 IU/L and range of 37–3198 IU/L) and bilirubin levels usually above 5 mg/dL (median of 5.8 mg/dL and range of 1–40 mg/dL) [80,83]. Treatment of AFLD by dedicated teams in intensive care units is mainly supportive until immediate delivery, preferably using a Cesarean section, in order to guarantee the best survival chances for the mother and the fetus [83,84].

## 4. Other Causes of Elevated Liver Enzymes: Hypotheses and Speculations

The true incidence of elevated LFTs during pregnancy is very difficult to establish because these tests are not routinely performed in all pregnant women and there is substantial heterogeneity among laboratories and centers regarding the definition of “normal” values of liver enzymes. However, some studies have shown that the prevalence of elevated LFTs is up to 5–13% [5,77,79,85], which is much higher than the canonic 3% prevalence of pregnancy-related liver disease [86], suggesting that there may be more to it than what was previously thought. Most studies have evaluated LFTs during the first trimester [77,85,87,88], showing that higher ALT levels are associated with an increased risk of future gestational diabetes [77,87,88], large-for-gestational-age babies [85,89,90], and pre-eclampsia [77,78,79]. In other studies, altered LFTs were investigated in the third trimester (or at delivery), with a reported prevalence of 11–13% [5]. Elevated LFTs were not always explained with pre-existing or pregnancy-related liver disease (30–50%) [5,91], and more importantly, could be associated with a higher risk of complications [5]. Finally, the incidence of NAFLD among pregnant women is rapidly increasing and is associated with an elevated risk of maternal complications and pre-term birth [92,93].

We hypothesize that the increasing incidence of NAFLD could explain the liver dysfunction in these patients, mainly through two synergic mechanisms: an unfavorable metabolic profile of the mother and a maladaptive response to the physiological circulatory changes that occur during pregnancy. First, NAFLD can manifest with elevated LFTs, and steatosis and insulin resistance associated with this condition could explain the increased risk of diabetes (as seen also in non-pregnant subjects) [94] and macrosomia (due to hyperinsulinism). On the other hand, maternal metabolic and cardiovascular dysfunction can lead to inadequate adaptation to the demands of pregnancy, with subsequent endothelial dysfunction driven by placental factors, finally leading to altered organ perfusion and venous congestion, including the liver [62,65,95,96]. This pathophysiological condition could create a continuum, and liver involvement could be present also in the absence of hypertension, as seen in some cases of HELLP syndrome [1,97]. In fact, a recent study [91] found significant changes in the splanchnic hemodynamics evaluated with Doppler ultrasound in patients with an undetermined cause of hypertransaminasemia when compared to controls, similar to what was seen in patients with pre-eclampsia [98]. Future studies aimed at evaluating the true prevalence of altered LFTs of an “unknown” origin, risk factors, and more importantly, association with maternal and fetal outcomes are eagerly awaited. Moreover, the role of the abdomen Doppler ultrasound and elastography [91,99,100] in the diagnostic work of these patients warrants further research.

## Figures and Tables

**Table 1:** Altered liver function tests in pregnancy-related liver disease.

	Hyperemesis Gravidarum	Intrahepatic Cholestasis of Pregnancy	PE-Related Liver Dysfunction and HELLP Syndrome	Acute Fatty Liver of Pregnancy
**Diagnostic Criteria**	Persistent vomiting with weight loss > 5%	Pruritus and bile acid > 10 µmol/L	Hypertension and maternal or fetal complication	Swansea criteria
**Altered liver enzymes**				
Prevalence	8–15% overall 40–50% if hospitalized	20–80%	20–50%	Almost 100%
Mean AST/ALT values (IU/L)	50 IU/L	100–150 IU/L	40–100 IU/L 300–500 IU/L if HELLP	300–1000 IU/L
Range (× ULN)	2–5 × ULN	1.5–8 × ULN	2–5 × ULN Up to 30 × ULN if HELLP	Up to 100 × ULN
**Jaundice**	Rare	Rare	Unlikely Possible (15%) if HELLP	Almost 100% Mean 5–6 mg/dL
**Inclusion in diagnostic criteria?**	No	No	Yes	Yes
**Correlation with disease severity and prognosis?**	Yes, with starvation	No prognostic role	Yes, with worse maternal outcomes	Unknown

ALT: alanine aminotransferase; AST: aspartate aminotransferase; HELLP: hemolysis, elevated liver enzymes, and low platelet syndrome; PE: pre-eclampsia; ULN: upper limit of normal.

## Data Availability

Data sharing not applicable.
